# Involvement of Glucocorticoids in the Reorganization of the Amphibian Immune System at Metamorphosis

**DOI:** 10.1155/1997/84841

**Published:** 1997

**Authors:** Louise A. Rollins-Smith, Katherine S. Barker, A. Tray Davis

**Affiliations:** Departments of Microbiology and Immunology and of Pediatrics Vanderbilt University School of Medicine Nashville Tennessee 37232-2580 USA

**Keywords:** Glucocorticoids, metamorphosis, lymphocytes, Xenopus

## Abstract

In recent years, integrative animal biologists and behavioral scientists have begun to understand the complex interactions between the immune system and the neuroendocrine system. Amphibian metamorphosis offers a unique opportunity to study dramatic hormone-driven changes in the immune system in a compressed time frame. In the South African clawed frog, *Xenopus laevis*, the larval pattern of immunity is distinct from that of the adult, and metamorphosis marks the transition from one pattern to the other. Climax of metamorphosis is characterized by significant elevations in thyroid hormones, glucocorticoid hormones, and the pituitary hormones, prolactin and growth hormone. Previously, we and others have shown that elevated levels of unbound glucocorticoid hormones found at climax of metamorphosis are associated with a natural decline in lymphocyte numbers, lymphocyte viability, and mitogen-induced proliferation. Here we present evidence that the mechanism for loss of lymphocytes at metamorphosis is glucocorticoid-induced apoptosis. Inhibition of lymphocyte function and loss of lymphocytes in the thymus and spleen are reversible by *in vitro* or *in vivo* treatment with the glucocorticoid receptor antagonist, RU486, whereas the mineralocorticoid receptor antagonist, RU26752, is poorly effective. These observations support the hypothesis that loss of larval lymphocytes and changes in lymphocyte function are due to elevated concentrations of glucocorticoids that remove unnecessary lymphocytes to allow for development of immunological tolerance to the new adult-specific antigens that appear as a result of metamorphosis.


*Developmental Immunology*, 1997, Vol. 5, pp. 145–152
Reprints available directly from the publisher
Photocopying permitted by license only

© 1997 OPA (Overseas Publishers Association)
Amsterdam B.V. Published under license in The Netherlands
under the Harwood Academic Publishers imprint
part of The Gordon and Breach Publishing Group
Printed in Malaysia

### Involvement of Glucocorticoids in the Reorganization of the Amphibian Immune System at Metamorphosis

LOUISE A. ROLLINS-SMITH*, KATHERINE S. BARKER, and A. TRAY DAVIS

*Departments of Microbiology and Immunology and of Pediatrics, Vanderbilt University School of Medicine, Nashville, Tennessee 37232-2580*

*(Received 26 November 1996)*

In recent years, integrative animal biologists and behavioral scientists have begun to understand the complex interactions between the immune system and the neuroendocrine system. Amphibian metamorphosis offers a unique opportunity to study dramatic hormone-driven changes in the immune system in a compressed time frame. In the South African clawed frog, *Xenopus laevis*, the larval pattern of immunity is distinct from that of the adult, and metamorphosis marks the transition from one pattern to the other. Climax of metamorphosis is characterized by significant elevations in thyroid hormones, glucocorticoid hormones, and the pituitary hormones, prolactin and growth hormone. Previously, we and others have shown that elevated levels of unbound glucocorticoid hormones found at climax of metamorphosis are associated with a natural decline in lymphocyte numbers, lymphocyte viability, and mitogen-induced proliferation. Here we present evidence that the mechanism for loss of lymphocytes at metamorphosis is glucocorticoid-induced apoptosis. Inhibition of lymphocyte function and loss of lymphocytes in the thymus and spleen are reversible by *in vitro* or *in vivo* treatment with the glucocorticoid receptor antagonist, RU486, whereas the mineralocorticoid receptor antagonist, RU26752, is poorly effective. These observations support the hypothesis that loss of larval lymphocytes and changes in lymphocyte function are due to elevated concentrations of glucocorticoids that remove unnecessary lymphocytes to allow for development of immunological tolerance to the new adult-specific antigens that appear as a result of metamorphosis.

*Keywords:* Glucocorticoids, metamorphosis, lymphocytes, *Xenopus*

### INTRODUCTION

Metamorphosis in amphibians presents a unique problem for the developing immune system. Free-living tadpoles are immunologically competent, but they acquire a new set of adult-specific molecules to which they must become immunologically tolerant at metamorphosis (reviewed in Du Pasquier, 1982; Cohen et al., 1985; Flajnik et al., 1987; Rollins-Smith and Cohen, 1996). How the metamorphosing tadpole avoids a self-destructive autoimmune response to these new molecules has intrigued immunologists for many years. One hypothesis is that unnecessary tadpole lymphocytes are discarded at metamorphosis and replaced

*Corresponding author.

by new lymphopoiesis after metamorphosis (Rollins-Smith and Blair, 1990a). In the South African clawed frog, *Xenopus laevis*, a dramatic loss of lymphocytes from the spleen, thymus, and liver (Du Pasquier and Weiss, 1973; Rollins-Smith et al., 1984; Cohen et al., 1985; Rollins-Smith et al., 1988) is associated with a significant increase in glucocorticoid hormones at metamorphic climax (Jaudet and Hatey, 1984; Jolivet-Jaudet and Leloup-Hatey, 1986). Viability and mitogen-stimulated proliferation of lymphocytes from metamorphosing frogs are reduced by concentrations of glucocorticoids naturally present at metamorphic climax (Marx et al., 1987; Rollins-Smith and Blair, 1993). Here, we report that the glucocorticoid receptor antagonist, RU486 (Moguilewsky and Philibert, 1984), can interfere with glucocorticoid-induced inhibition of proliferation and glucocorticoid-induced apoptosis of lymphocytes. Moreover, RU486, delivered to tadpoles in their water at metamorphic climax, results in increased numbers of lymphocytes recovered from spleen and thymus. These findings suggest that naturally elevated glucocorticoids at metamorphic climax induce the death of some lymphocytes and may inhibit the function of others, allowing for the acceptance of new adult-specific molecules by the immune system.

### RESULTS

### Inhibition of Lymphocyte Proliferation by Glucocorticoids Is Reversed by RU486

In *X. laevis*, like other amphibians, the major glucocorticoids produced are corticosterone and aldosterone (Chan and Edwards, 1970; Jolivet-Jaudet and Leloup-Hatey, 1984). Plasma concentrations of unbound corticosterone and aldosterone are elevated at climax of metamorphosis to a maximum of about 70 and 30 nM, respectively (Jaudet and Hatey, 1984; Jolivet-Jaudet and Leloup-Hatey, 1986). Proliferation of splenic lymphocytes from metamorphosing frogs driven by phytohemagglutinin-P (PHA) is significantly reduced by each hormone at a concentration of 1–10 nM (Fig. 1). Addition of a ten- to 100-fold molar excess of RU486 to such PHA-stimulated cultures interferes with the glucocorticoid-induced inhibition of proliferation (Fig. 1). These experiments demonstrate that a glucocorticoid-induced effect (inhibition of proliferation) is reversible in the presence of RU486. The fact that the effects of both corticosterone and aldosterone are antagonized by RU486 suggests that both hormones mediate their effects through engagement of the glucocorticoid receptor. This is supported by the observation that a specific mineralocorticoid receptor antagonist,

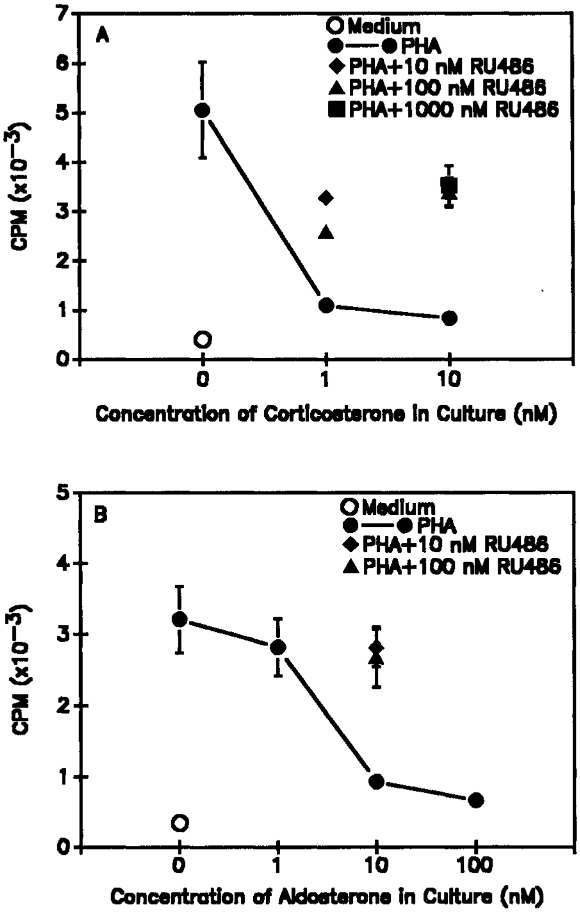

FIGURE 1 Inhibition of splenic lymphocyte proliferation by glucocorticoids reversed by RU486. Lymphocytes, removed from tadpoles (prometamorphic stages 55–56) of the MHC homozygous J-Strain (Tochinai and Katagiri, 1975; DiMarzo and Cohen, 1982), were cultured as described in Materials and Methods. PHA-induced [$^3$H]TdR uptake was significantly inhibited by addition of 1 or 10 nM corticosterone (panel A) or 10, or 100 nM aldosterone (panel B) (one-tailed Student's *t*-test; $p \leq 0.01$). RU486 (a gift from Roussel Uclaf, Romainville, France), added at a concentration of 10, 100, or 1000 nM 2 hr before the addition of corticosterone or aldosterone significantly reversed the glucocorticoid inhibition (one-tailed Student's *t*-test; $p \leq 0.005$). Four to six replicate cultures were plated for each parameter tested. Results are presented as mean ± standard error counts per minute (CPM). The experiments presented in panels A and B are representative of seven and four replicate experiments, respectively.

RU26752, (Nédélec et al., 1985) is a poor inhibitor of the aldosterone-mediated effects on proliferation (Fig. 2). In the experiment shown, RU486, at concentrations of 10 or 100 nM, significantly reversed the effects of 10 nM aldosterone (one-tailed Student's *t*-test; $p \leq 0.005$), whereas only the highest concentration (1000 nM) of RU26752 significantly reversed the effect (one-tailed Students *t*-test; $p \leq 0.01$).

### Corticosterone-Induced Apoptosis Is Reversed by RU486 in Culture

In addition to their effects on proliferation, corticosteroids are known to induce the death of thymocytes and peripheral lymphocytes (Wyllie, 1980; Rollins-Smith and Blair, 1993) by the process of programmed cell death or apoptosis (Wyllie, 1980). Freshly isolated splenocytes from metamorphosing frogs or those splenocytes cultured in medium alone for 24 hr at 4°C show a limited amount of apoptotic cell death measurable as nuclei with a hypodiploid complement of DNA (2.4%; Fig. 3A). Culture in medium alone at 26°C results in an increased level of spontaneous apoptosis (24.4%; Fig. 3B) that is further increased in the presence of 10 nM corticosterone (47.9%; Fig. 3C). Addition of RU486 reduces the corticosterone-induced apoptosis to the level of background spontaneous apoptosis (24.8%; Fig. 3D), whereas RU486 alone does not induce increased apoptosis above the level observed in the medium control (23.0%; Fig. 3E). Together, these *in vitro* effects of RU486 demonstrate that it is an effective antagonist of glucocorticoid-induced changes in the amphibian immune system.

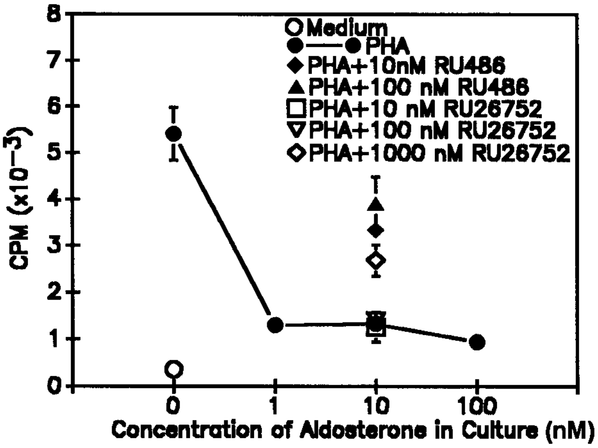

FIGURE 2 Inhibition of splenic lymphocyte proliferation effectively reversed by RU486 but poorly reversed by RU26752. PHA-induced [$^3$H]TdR uptake was significantly reduced by addition of 1, 10, or 100 nM aldosterone (one-tailed Student's *t*-test; $p \leq 0.005$). The effects of addition of 10 or 100 nM RU486 or 10, 100, or 1000 nM RU26752 are indicated by the appropriate symbols. Results are presented as mean ± standard error CPM. The experiment presented is representative of three replicate experiments

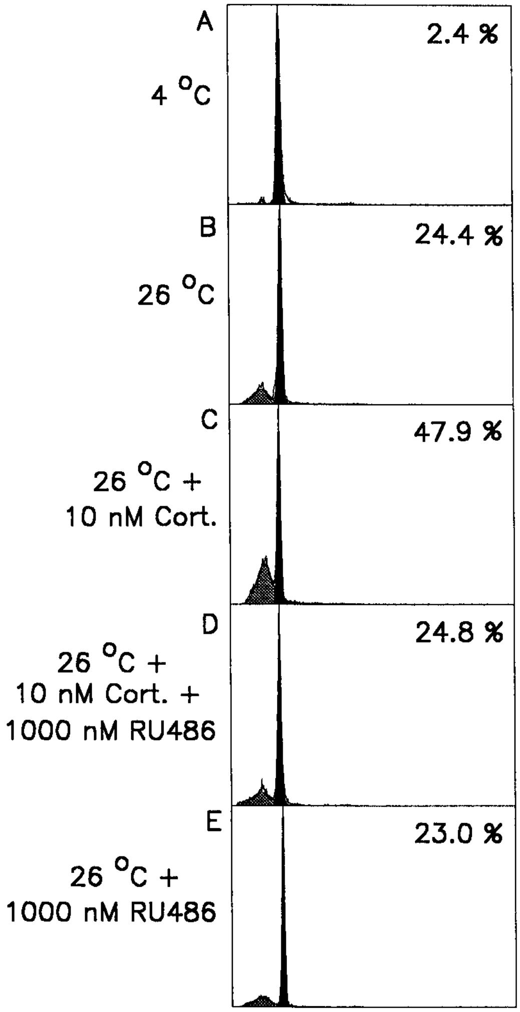

FIGURE 3 Spontaneous and corticosterone-induced apoptosis reversed by RU486. Splenocytes, pooled from three J-strain frogs at stages 63–64 of metamorphic climax, were incubated for 24 hr at 4°C or 26°C with or without 10 nM corticosterone and with or without 1000 nM RU486, as shown. Following fixation and rehydration, they were stained with propidium iodide and analyzed for nuclei with hypodiploid DNA (hatched peak) by flow cytometry as described in Materials and Methods. The percentage of cells with apoptotic nuclei is shown in the upper right of each panel. This experiment is representative of nine similar experiments done with splenocytes from metamorphosing frogs.

### Delivery of RU486 by Immersion Inhibits Lymphocyte Cell Loss from the Spleen and Thymus of Metamorphosing Frogs

To determine whether glucocorticoids play a significant role in the loss of lymphocytes *in vivo*, tadpoles, at advanced prometamorphic stages (stages 57–58 of Nieuwkoop and Faber, 1967), were immersed in variable concentrations of RU486 or vehicle only and treated continuously until they were sacrificed at metamorphic climax stages 64–65. These are the stages at which splenocyte and thymocyte numbers reach their lowest point during metamorphic transition (Du Pasquier and Weiss, 1973; Rollins-Smith et al., 1984; Cohen et al., 1985). Total recoverable lymphocytes in the thymus and spleen were significantly increased by RU486 treatment in a dose-dependent fashion (Fig. 4). These findings, therefore, argue strongly that antagonism of glucocorticoid effects *in vivo*, as well as *in vitro*, can result in increased survival of lymphocyte populations in the thymus and spleen.

### Effects of RU486 on Wet Weight and the Rate of Progression through Metamorphosis

In addition to the observed effects on lymphocyte numbers in the thymus and spleen, we observed a dose-dependent increase in wet weight of RU486-treated frogs (Fig. 5). Weight was significantly increased at concentrations of 15 or 150 nM RU486 in the water (one-tailed Student's *t*-test; $p \leq 0.005$). We also observed that treatment with the highest concentration of RU486 (150 nM) resulted in a significant delay in progression of metamorphosis to stages 64–65. Vehicle-treated control frogs reached stages 64–65 in 13.9 ± 1.0 days, whereas those treated with 150 nM RU486 required 18.6 ± 1.8 days (Fig. 6).

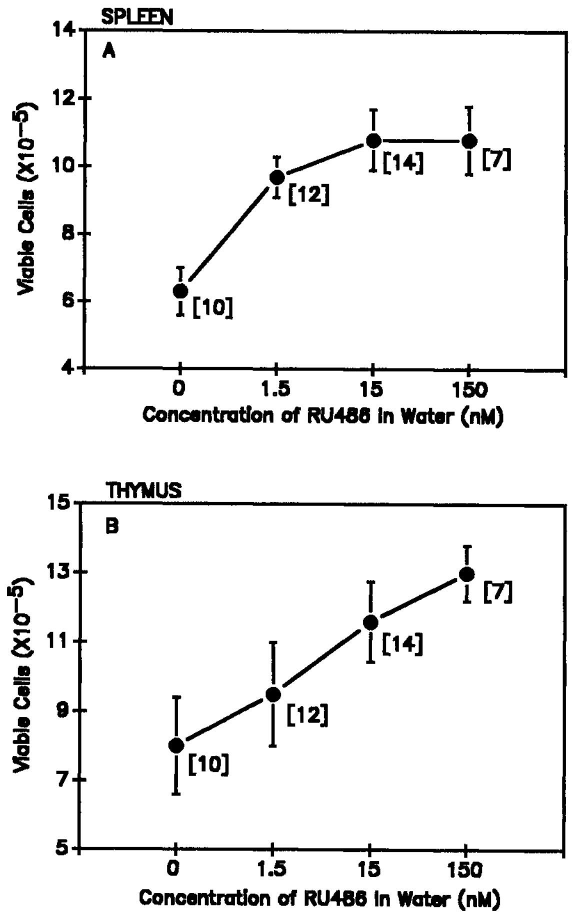

FIGURE 4 Effects of *in vivo* treatment with RU486 on lymphocyte cell loss from the spleen (panel A) and thymus (panel B) of metamorphosing frogs. Stage 57–58 J-Strain tadpoles were immersed in dechlorinated tap water with or without RU486 as described in Materials and Methods. All frogs were sacrificed when they reached metamorphic stages 64–65. Viable thymocytes and splenocytes were enumerated as described previously (Rollins-Smith and Blair, 1990b). Brackets indicate the number of frogs in each experimental group (pooled from three identical experiments). RU486 treatment significantly increased the number of recoverable splenocytes at all concentrations tested (one-tailed Student's *t*-test; $p \leq 0.005$) and increased the number of recoverable thymocytes at 15 and 150 nM concentrations (one-tailed Student's *t*-test; $p \leq 0.05$).

### DISCUSSION

These studies demonstrate the effectiveness of RU486 in this amphibian model system. Inhibition of proliferation and induction of apoptosis are reversed *in vitro*; and *in vivo* treatment results in inhibition of lymphocyte loss, an increase in wet weight, and a delay in progression through metamorphosis. Elevated levels of glucocorticoids at metamorphosis are thought to be involved in the mobilization of energy reserves necessary for tissue remodeling at a time of fasting (Hanke and Leist, 1971; Jaudet and Hatey, 1984). A number of thyroid hormone-driven events during climax of metamorphosis are accelerated by glucocorticoids (see reviews in Kikuyama et al., 1993; and Kaltenbach, 1996). Thus,

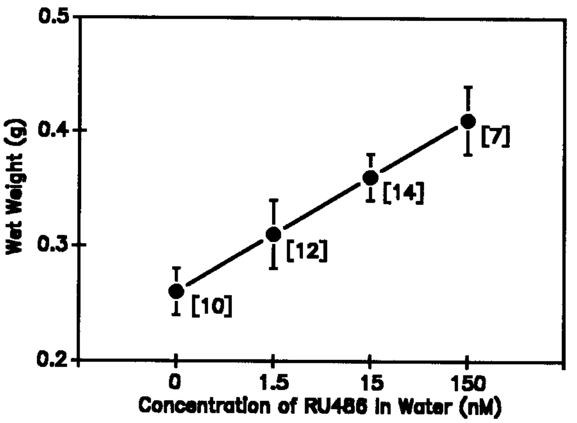

FIGURE 5 Effects of immersion in RU486 on wet weight. Brackets indicate the number of individuals in each experimental group (pooled from three identical experiments). The weight was significantly increased by immersion in 15 or 150 nM RU486 (one-tailed Student's $t$-test; $p \leq 0.005$).

it may not be surprising that inhibition of glucocorticoid receptor binding by RU486 has the general effect of delaying metamorphic progression. Our results are consistent with the previous observation of Kikuyama and co-workers that treatment of metamorphosing tadpoles with an agent that inhibits corticosteroid synthesis (Amphenone B) delays resorption of the tail (Kikuyama et al., 1982). The increase in wet weight following RU486 treatment is thought to be due to increased water retention. This RU486 effect is something of a puzzle. We speculate that it may be due to interference with pituitary hypothalamic feedback mechanisms that results in increased levels of both corticosterone and aldosterone. The function of mineralocorticoid receptors (type I) would be largely intact because RU486 binds poorly to them (Garty et al., 1994), whereas the function of glucocorticoid (type II) receptors would be specifically blocked (Garty et al., 1994). Previous studies have shown that normally metamorphosing frogs lose significant amounts of water during climax stages 59–63 (Hanke and Leist, 1971). RU486 treatment might have the net effect of increasing both corticosterone and aldosterone levels, resulting in fluid retention.

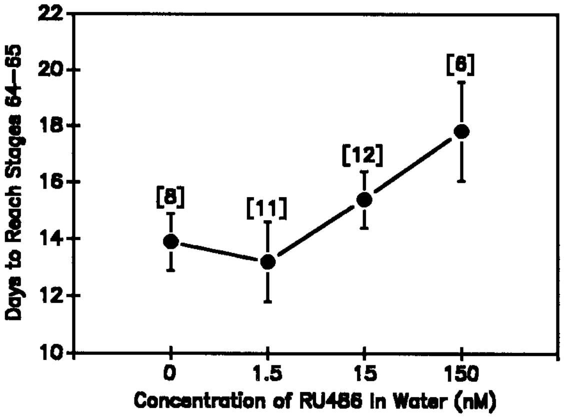

FIGURE 6 Effects of immersion in RU486 on progression through metamorphosis. The number of days from the beginning of treatment until sacrifice at stages 64–65 are plotted. Brackets indicate the number of animals in each group (pooled from two identical experiments). Metamorphosis was significantly delayed by treatment with 150 nM RU486 (Student's $t$-test; $p \leq 0.05$).

Tadpole lymphocytes are expendable and potentially deleterious to the adult. Metamorphosis is accompanied by the appearance of a variety of new molecules and structures unique to the adult stage of life, including adult hemoglobin (Just et al., 1977, 1980; Widmer et al., 1981; reviewed in Weber, 1996), adult-type keratin (Ellison et al., 1985; Mathisen and Miller, 1987; 1989; reviewed in Miller, 1996), the urea cycle enzymes (Morris, 1987; Galton et al., 1991; Helbing et al., 1992; Xu et al., 1993; Atkinson, 1994; Helbing and Atkinson, 1994; reviewed in Atkinson et al., 1996), and vitellogenin (Wahli et al., 1981; Knowland, 1985). Persisting larval lymphocytes theoretically could develop a destructive autoimmune response to these new antigens. Previous studies from my laboratory have sought to understand to what extent larval lymphocytes persist in the postmetamorphic period. Clearly, some lymphocytes can persist because immunological memory persists through metamorphosis (Du Pasquier and Haimovich, 1976; Barlow and Cohen, 1983; Cohen et al., 1985; Manning and Al Johari, 1985). Using triploid (3N) thymus implants in diploid (2N) hosts, we have demonstrated that although some larval T lymphocytes persist, most are naturally deleted or significantly diluted by the expanding adult population (Rollins-Smith et al., 1992). Additional studies examined the fate of larval T cells in individuals thymectomized just prior to metamorphosis. These experiments also showed that a small population of larval T cells persists after metamorphosis, although T lymphocyte numbers were dramatically reduced by thymectomy (Rollins-Smith et al., 1996). Because RU486 treatment reduces the number of lymphocytes lost at metamorphosis, it is now possible to study whether abnormally persisting larval lymphocytes can respond to adult-specific antigens. Future studies will address this question.

Loss of lymphocytes by untreated frogs may temporarily expose metamorphosing frogs to a greater risk of attack by environmental pathogens. Studies are currently planned to determine whether metamorphosing frogs are, indeed, more susceptible to infection by pathogens than younger larvae or postmetamorphic adults. If metamorphosing frogs survive the temporary immunosuppression, the immune system undergoes a period of renewal and expansion that results in a more extensive antigen-recognition repertoire and immunological tolerance to newly emerging adult-specific molecules (reviewed in Du Pasquier, 1982; Cohen, et al., 1985; Flajnik et al., 1987; Rollins-Smith and Cohen, 1996). The removal of unnecessary tadpole lymphocytes by glucocorticoids seems to be an exquisite example of the integration of the immune system and the endocrine system that benefits the organism.

### MATERIALS AND METHODS

### Frogs

*Xenopus laevis* J-Strain (Tochinai and Katagiri, 1975; DiMarzo and Cohen, 1982), which are homozygous at the major histocompatibility complex locus, were used for all of the experiments reported here. Adult males and females were induced to breed by injection of human chorionic gonadotropin according to standard procedures as described previously (Rollins-Smith and Blair, 1990b). Larvae were reared at approximately 8–10 tadpoles per 4 liters of dechlorinated tap water. Water was changed and animals were fed powdered nettle leaf three times weekly. Larval stages were determined according to the Normal Table of Nieuwkoop and Faber (1967). All animals used in these experiments were cared for by trained technicians in accordance with Vanderbilt University institutional guidelines.

### Lymphocyte Proliferation Assay

Larval splenocytes were prepared and cultured as described previously (Rollins-Smith et al., 1984). Briefly, dissociated cells were cultured at a density of $1–1.25 \times 10^5$/ml in Leibovitz (L-15) culture medium (GIBCO, Grand Island, NY) diluted to amphibian tonicity and supplemented with 100 units/ml penicillin, 100 μg/ml streptomycin, $1.25 \times 10^{-2}$ M sodium bicarbonate, $5 \times 10^{-5}$ M 2-mercaptoethanol, and 1% heat-inactivated fetal calf serum (complete medium). Proliferation of T lymphocytes was induced by addition of phytohemagglutinin-P (PHA) at a concentration of 2 μg/ml. Cultures were assayed for PHA-induced [$^3$H]-thymidine ([$^3$H]TdR) incorporation according to published procedures (Rollins-Smith et al., 1984). Briefly, after incubation at 26°C for 48 h in a 5% $CO_2$-95% air atmosphere, the cultures were pulsed with 0.5 μCi of [$^3$H]TdR (5 μCi/ml; specific activity = 2 Ci/mmole) (New England Nuclear, Boston) and incubated for an additional 24 h before harvesting. Four to six replicate cultures were plated for each parameter tested. Results are presented as mean ± standard error counts per minute (CPM).

### Steroid Hormones and Antagonists

Corticosterone and aldosterone were purchased from Sigma (St. Louis). RU486 and RU26752 were generous gifts from Roussel Uclaf (Romainville, France). All hormones and antagonists were diluted to the appropriate concentration in complete medium from freshly prepared stock solutions made up at $10^{-2}$ M in 95% ethanol.

### Apoptosis Assay

Splenocytes were incubated for 24 h at 4°C or 26°C with or without 10 nM corticosterone and with or without 1000 nM RU486. Following fixation in ethanol (9 parts 70% ethanol, 1 part amphibian phosphate-buffered saline) (APBS) for 24 h at − 20°C, they were rehydrated in APBS, stained with propidium iodide (2 mg per 100 ml of APBS + 0.1 ml Triton X-100 + 3.7 mg EDTA), and treated with RNAse according to the method of Hotz and co-workers (1994) modified by our laboratory as described here for amphibian cells. Percent apoptotic nuclei exhibiting a hypodiploid DNA content were enumerated by flow cytometry using a Becton-Dickenson FACSTAR. Data collected in the list mode were analyzed using the MODFIT analysis program (Verity Software House, Topsham, ME).

### *Treatment of Frogs with RU486 by Immersion*

Stage 57–58 J-strain tadpoles were immersed in dechlorinated tap water alone or with increasing concentrations of RU486. A stock solution of RU486 at $10^{-2}$ M in 95% ethanol was further diluted to $1.5 \times 10^{-3}$ M with a 1:1 mixture of propylene glycol and distilled water. One hundred, 10, or 1 µl were added per liter of frog water to achieve a concentration of 1.5, 15, or 150 nM RU486 in the water. Control frogs received 100 µl per liter of the vehicle alone. The water was changed and fresh hormone was added three times weekly. All frogs were sacrificed when they reached metamorphic stages 64–65, and the number of days from the beginning of treatment was recorded for each individual.

### *Determination of Wet Weight*

At the time of sacrifice, RU486-treated frogs and controls were blotted dry and weighed to the nearest 0.1 gram with a metric balance.

### *Acknowledgements*

This work was supported by grants DCB-9004666 and MCB-9421349 from the National Science Foundation (to L.R.-S.) and grant HD05797 from the NIH made to the Vanderbilt Center for Reproductive Biology Research (M-C Orgebin-Crist, P.I.). We thank W. Green and J. Price of the flow cytometry laboratory of the Veterans Administration Medical Center of Nashville for assistance with flow cytometry. RU486 was a generous gift of Roussel Uclaf, Romainville, France.

### *References*

Atkinson, B. G. (1994). Metamorphosis: Model systems for studying gene expression in postembryonic development. *Dev. Genet.* **15**: 313–319.

Atkinson, B. G., Helbing, C., and Chen, Y. (1996). Reprogramming of genes expressed in amphibian liver during metamorphosis. *In*: Metamorphosis: Postembryonic Reprogramming of Gene Expression in Amphibian and Insect Cells, Gilbert L. I., Tata J. R., and Atkinson B. G., Eds. (New York: Academic Press), pp. 539–566.

Barlow, E. H., and Cohen, N. (1983). The thymus dependency of transplantation allotolerance in the metamorphosing frog, *Xenopus laevis*. *Transplantation* **35**: 612–619.

Chan, S. T. H., and Edwards, B. R. (1970). Kinetic studies on the biosynthesis of corticosteroids *in vitro* from exogenous precursors by the interrenal glands of the normal, corticotrophin-treated, and adenohypophysectomized *Xenopus laevis* Daudin. *J. Endocrinol.* **47**: 183–195.

Cohen, N., DiMarzo, S., Rollins-Smith, L., Barlow, E., and Vanderschmidt-Parsons, S. (1985). The ontogeny of allo-tolerance and self-tolerance in larval *Xenopus laevis*. *In*: Metamorphosis, Balls M., and Bownes M., Eds. (Oxford: Oxford University Press), pp. 388–419.

DiMarzo, S., and Cohen, N. (1982). Immunogenetic aspects of *in vivo* allotolerance induction during ontogeny of *Xenopus laevis*. *Immunogenetics* **16**: 103–116.

Du Pasquier, L. (1982). Ontogeny of immunological functions in amphibians. *In*: The Reticuloendothelial System, A Comprehensive Treatise, Vol. 3: Phylogeny and Ontogeny, Cohen N., and Sigel M. M., Eds. (New York: Plenum Press), pp. 633–657.

Du Pasquier, L., and Haimovich, J. (1976). The antibody response during amphibian ontogeny. *Immunogenetics* **3**: 381–391.

Du Pasquier, L., and Weiss, N. (1973). The thymus during the ontogeny of the toad *Xenopus laevis*: Growth, membrane-bound immunoglobulins and mixed lymphocyte reaction. *Eur. J. Immunol.* **3**, 773–777.

Ellison, T. R., Mathisen, P. M., and Miller, L. (1985). Developmental changes in keratin patterns during epidermal maturation. *Dev. Biol.* **112**: 329–337.

Flajnik, M. F., Hsu, E., Kaufman, J. F., and Du Pasquier, L. (1987). Changes in the immune system during metamorphosis of *Xenopus. Immunol.* Today **8**: 58–64.

Galton, V. A., Morganelli, C. M., Schneider, M. J., and Yee, K. (1991). The role of thyroid hormone in the regulation of hepatic carbamyl phosphate synthetase activity in *Rana catesbeiana*. *Endocrinology* (Baltimore) **129**: 2298–2304.

Garty, H., Peterson-Yantorno, K., Asher, C., and Civan, M. M. (1994). Effects of corticoid agonists and antagonists on apical $Na^+$ permeability of toad urinary bladder. *Amer. J. Physiol.* **266**: F108–F116.

Hanke, W., and Leist, K. H. (1971). The effect of ACTH and corticosteroids on carbohydrate metabolism during the metamorphosis of *Xenopus laevis*. *Gen. Comp. Endocrinol.* **16**: 137–148.

Helbing, C. C., and Atkinson, B. G. (1994). 3,5,3′-Triiodothyronine-induced carbamyl phosphate synthetase gene expression is stabilized in the liver of *Rana catesbeiana* tadpoles during heat shock. *J. Biol. Chem.* **269**: 11743–11750.

Helbing, C. C., Gergely, G., and Atkinson, B. G. (1992). Sequential up-regulation of thyroid hormone β receptor, ornithine transcarbamylase, and carbamyl phosphate synthetase mRNAs in the liver of *Rana catesbeiana* tadpoles during spontaneous and thyroid hormone-induced metamorphosis. *Dev. Genet.* **13**: 289–301.

Hotz, M. A., Gong, J., Traganons, F., and Darzynkiewicz, Z. (1994). Flow cytometric detection of apoptosis: Comparison of the assays of *in situ* DNA degradation and chromatin changes. *Cytometry* **15**: 237–244.

Jaudet, G. J., and Hatey, J. H. (1984). Variations in aldosterone and corticosterone plasma levels during metamorphosis in *Xenopus laevis* tadpoles. *Gen. Comp. Endocrinol.* **56**: 59–65.

Jolivet-Jaudet, G., and Leloup-Hatey, J. (1984). Interrenal function during amphibian metamorphosis: *In vitro* biosynthesis of radioactive corticosteroids from ($4^{14}C$)-progesterone by interrenal in *Xenopus laevis* tadpoles. *Comp. Biochem. Physiol.* **79B**: 239–244.

Jolivet-Jaudet, G., and Leloup-Hatey, J. (1986). Corticosteroid binding in plasma of *Xenopus laevis*. Modifications during metamorphosis and growth. *J. Steroid Biochem.* **25**: 343–350.

Just, J. J., Schwager, J., and Weber, R. (1977). Hemoglobin transition in relation to metamorphosis in normal and isogenic *Xenopus*. *Wilhelm Roux's Arch. Dev. Biol.* **183**: 307–323.

Just, J. J., Schwager, J., Weber, R., Fey, H., and Pfister, H. (1980). Immunological analysis of hemoglobin transition during metamorphosis of normal and isogenic *Xenopus*. *Wilhelm Roux's Arch. Dev. Biol.* **188**: 75–80.

Kaltenbach, J. C. (1996). Endocrinology of amphibian metamorphosis. *In:* Metamorphosis: Postembryonic Reprogramming of Gene Expression in Amphibian and Insect Cells, Gilbert L. I., Tata J. R., and Atkinson B. G., Eds. (New York: Academic Press), pp. 403–431.

Kikuyama, S., Kawamura, K., Tanaka, S., and Yamamoto, K. (1993). Aspects of amphibian metamorphosis: Hormonal control. Int. Rev. Cytol. **145**: 105–148.

Kikuyama, S., Niki, K., Mayumi, M., and Kawamura, K. (1982). Retardation of thyroxine-induced metamorphosis by Amphenone B in toad tadpoles. *Endocrinol. Jpn.* **29**: 659–662.

Knowland, J. (1985). Vitellogenesis and levels of oestrogen receptor in *Xenopus* liver during metamorphosis. *In:* Metamorphosis, Balls M., and Bownes M., Eds. (Oxford: Oxford University Press), pp. 108–116.

Manning, M. J., and Al Johari, G. M. (1985). Immunological memory and metamorphosis. *In:* Metamorphosis, Balls M., and Bownes M., Eds. (Oxford: Oxford University Press), pp. 420–433.

Marx, M., Ruben, L. N., Nobis, C., and Duffy, D. (1987). Compromised T-cell regulatory functions during anuran metamorphosis: The role of corticosteroids. *In:* Developmental and Comparative Immunology, Cooper E. L., Langlet C., and Bierne J., Eds. (New York: Alan R. Liss), pp. 129–140.

Mathisen, P. M., and Miller, L. (1987). Thyroid hormone induction of keratin genes: A two-step activation of gene expression during development. *Genes Dev.* **1**: 1107–1117.

Mathisen, P. M., and Miller, L. (1989). Thyroid hormone induces constitutive keratin gene expression during *Xenopus laevis* development. *Mol. Cell Biol.* **9**: 1823–1831.

Miller, L. (1996). Hormone-induced changes in keratin gene expression during amphibian skin metamorphosis. *In:* Metamorphosis: Postembryonic Reprogramming of Gene Expression in Amphibian and Insect Cells, Gilbert L. I., Tata J. R., and Atkinson B. G., Eds. (New York: Academic Press), pp. 599–624.

Moguilewsky, M., and Philibert, D. (1984). RU38486: Potent antiglucocorticoid activity correlated with strong binding to the cytosolic glucocorticoid receptor followed by an impaired activation. *J. Steroid Biochem.* **20**: 271–276.

Morris, S. M. Jr. (1987). Thyroxine elicits divergent changes in mRNA levels for two urea cycle enzymes and one gluconeogenic enzyme in tadpole liver. *Arch. Biochem. Biophys.* **259**: 144–148.

Nédélec, L., Philibert, D., and Torelli, V. (1985). Recent developments in the field of steroid antihormones. *In:* Third SCI-RSC Medicinal Chemistry Symposium, Lambert R. W., Ed. (London: Royal Society of Chemistry), pp. 322–344.

Nieuwkoop, P. D., and Faber, J. (1967). Normal Table of *Xenopus laevis (Daudin)* (Amsterdam: North Holland).

Rollins-Smith, L. A., and Blair, P. (1990a). Expression of class II major histocompatibility complex antigens on adult T cells in *Xenopus* is metamorphosis dependent. *Dev. Immunol.* **1**: 97–104.

Rollins-Smith, L. A., and Blair, P. (1990b). Contribution of ventral blood island mesoderm to hematopoiesis in postmetamorphic and metamorphosis-inhibited *Xenopus laevis*. *Dev. Biol.* **142**: 178–183.

Rollins-Smith, L. A., and Blair, P. J. (1993). The effects of corticosteroid hormones and thyroid hormones on lymphocyte viability and proliferation during development and metamorphosis of *Xenopus laevis*. Differentiation (Berlin) **54**: 155–160.

Rollins-Smith, L. A., Blair, P. J., and Davis, A. T. (1992). Thymus ontogeny in frogs: T cell renewal at metamorphosis. *Dev. Immunol.* **2**: 207–213.

Rollins-Smith, L. A., and Cohen, N. (1996). Metamorphosis: An immunologically unique period in the life of the frog. *In:* Metamorphosis: Postembryonic Reprogramming of Gene Expression in Amphibian and Insect Cells, Gilbert L. I., Tata J. R., and Atkinson B. G., Eds. (New York: Academic Press), pp. 625–646.

Rollins-Smith, L. A., Needham, D. A. P., Davis, A. T., and Blair, P. J. (1996). Late thymectomy in *Xenopus* tadpoles reveals a population of T cells that persists through metamorphosis. *Dev. Comp. Immunol.* **20**: 165–174.

Rollins-Smith, L. A., Parsons, S. C. V., and Cohen, N. (1984). During frog ontogeny, PHA and Con A responsiveness of splenocytes precedes that of thymocytes. *Immunology* **52**: 491–500.

Rollins-Smith, L. A., Parsons, S. C. V., and Cohen, N. (1988). Effects of thyroxine-driven precocious metamorphosis on maturation of adult-type allograft rejection responses in early thyroidectomized frogs. Differentiation **37**: 180–185.

Tochinai, S., and Katagiri, C. (1975). Complete abrogation of immune response to skin allografts and rabbit erythrocytes in the early thymectomized *Xenopus*. *Dev. Growth Diff.* **17**: 383–394.

Wahli, W., Dawid, I. B., Ryffel, G. U., and Weber, R. (1981). Vitellogenesis and the vitellogenin gene family. *Science* **212**: 298–304.

Weber, R. (1996). Switching of globin genes during anuran metamorphosis. *In*: Metamorphosis: Postembryonic Reprogramming of Gene Expression in Amphibian and Insect Cells, Gilbert L. I., Tata J. R., and Atkinson B. G., Eds. (New York: Academic Press), pp. 567–597.

Widmer, H. J., Andres, A.-C., Niessing, J., Hosbach, H. A., and Weber, R. (1981). Comparative analysis of cloned larval and adult globin cDNA-sequences of *Xenopus laevis*. *Dev. Biol.* **88**: 325–332.

Wyllie, A. H. (1980). Glucocorticoid induced thymocyte apoptosis is associated with endogenous endonuclease activation. *Nature* **284**: 555–556.

Xu, Q., Baker, B. S., and Tata, J. R. (1993). Developmental and hormonal regulation of *Xenopus* liver-type arginase gene. *Eur. J. Biochem.* **211**: 891–898.

